# Nucleotide Excision Repair Factor XPC Ameliorates Prognosis by Increasing the Susceptibility of Human Colorectal Cancer to Chemotherapy and Ionizing Radiation

**DOI:** 10.3389/fonc.2018.00290

**Published:** 2018-07-31

**Authors:** Liang-Bo Hu, Yin Chen, Xiao-Dong Meng, Pan Yu, Xu He, Jie Li

**Affiliations:** ^1^Department of Radiology, Yongchuan Hospital of Chongqing Medical University, Chongqing, China; ^2^Department of General Surgery, The People's Liberation Army 324 Hospital, Chongqing, China; ^3^Department of Urology, Bethune International Peace Hospital, Shijiazhuang, China; ^4^Department of Burn and Plastic Surgery, Jinling Hospital, Nanjing, China; ^5^Department of Nephrology, Yongchuan Hospital of Chongqing Medical University, Chongqing, China

**Keywords:** chemotherapy, colorectal neoplasms, nucleotide excision repair (NER), prognosis, XPC

## Abstract

Nucleotide excision repair (NER) is a DNA damage repair mechanism in mammals, but the relationship between NER and human colorectal cancer (HRC) progression has not been clarified yet. In this study, the expression of the NER genes XPA, XPC, XPF, XPG, ERCC1, and XPD was measured in normal and cancerous human colorectal tissue. Among them, only the XPC gene expression was significantly increased in colorectal cancer tissue. To establish the role of XPC in colorectal cancer, small interference RNA (siRNA) targeting XPC was used to knockdown the expression of XPC in HRC cell lines. In addition, an expression vector plasmid containing the XPC cDNA was constructed and stably transfected into HRC cell lines to overexpress the XPC gene. Interestingly, MTT and apoptosis assay demonstrated that XPC gene overexpression significantly increased the susceptibility of HRC cell lines to cisplatin and X-ray radiation. In order to study the relationship between XPC expression and the progression of HRC, XPC expression was measured in 167 patients with colorectal cancer. The results showed that patients with high XPC expression had longer survival time. Cox regression analysis showed that high XPC expression might be a potential predictive factor for colorectal cancer. In conclusion, XPC plays a key role in the susceptibility of colorectal cancer to chemotherapy and ionizing radiation and is associated with a good patients' prognosis.

## Introduction

DNA repair plays an important role in maintaining genome integrity in both normal and tumor cells (1, 2). Since many chemotherapeutic agents cause DNA damage, the abnormal expression of DNA damage repair genes is closely related to tumor prognosis (3, 4). Among various DNA repair pathways, nucleotide excision repair (NER) is the main defensive barrier against DNA damage (5, 6) and a major repair system for chemo radiotherapy-induced DNA damage (7, 8).

NER works via two pathways: global genome repair (GGR) and transcription-coupled repair (TCR) (9, 10). GGR is involved in injury repair for any genomic sequence, which is important to prevent carcinogenesis. The most important TCR function is to repair the DNA damage of actively transcriptional chains, which may be associated with tumor chemosensitivity. Monger proteins, xeroderma pigmentosum complementation group C (XPC) and its accessory subunits have been identified as protein complexes involved in recognizing DNA lesions and consequent recruit of other repair proteins (11). XPC defects are linked with many cancer types (12–14). Besides XPC, xeroderma pigmentosum gene group A (XPA) is another important NER gene, which works at the intersecting step of GGR and TCR pathways (15). Neither GGR nor TCR is initiated in the absence of XPA. Xeroderma pigmentosum group F (XPF) combines with ERCC1 to form a dimer that works as a 5′ DNA endonuclease, and XPD and XPG work as a DNA ligase and a 3′ DNA endonuclease (16), respectively. Although the NER mechanism involves approximately 30 proteins, these proteins are involved in the initiation stage of NER, and are its core proteins. However, the precise role of these NER proteins in colorectal cancer remains unclear.

Colorectal cancer is the second-most and third-most common cancer in women and men, respectively (17). Surgery and chemotherapy or radiotherapy are the main treatments for colorectal cancer. The 5 year overall survival rate of patients with colorectal cancer is approximately 50–75%, but the 3 year survival rate of patients with stage III colon cancer is only around 60% (18–20). Resistance to chemotherapy or radiotherapy reduces the survival time of patients with colorectal cancer (21, 22). The mechanism involved in the chemo radiotherapy resistance is not yet clear. Previous studies reported that the polymorphism of NER genes is associated with the prognosis of colorectal cancer (23–26), but the relationship between NER and colorectal cancer drug resistance is still poorly documented.

In the present study, the expression of NER genes (XPC, XPA, XPG, XPF, ERCC1, and XPD) was measured in human colorectal cancer and the corresponding normal tissues. The role of these genes in colorectal cancer susceptibility to chemotherapy and radiotherapy was also evaluated. In addition, the expression of these genesin colorectal cancer tissues from167 patients was evaluated by the Kaplan-Meier method and Cox proportional hazards regression. Based on these analyses, our aim was to clarify the relationship between NER genes and colorectal cancer prognosis, to provide some evidences on the role of targeting differential genes in colorectal cancer clinical treatment.

## Materials and methods

### Clinical data and specimen collection

All patients recruited in the present study read and signed the informed consent form, and the study was approved by the Ethics Committee of PLA324 Hospital and Bethune International Peace Hospital.

The clinical trial was divided into two separate sections. The first section included 36 fresh colorectal cancer specimens and 14 specimens of adjacent normal tissues that were collected from patients (25 males and 11 females, average age 52.5 ± 16.6 years) recruited between October 2009 and May 2010 at the Department of General Surgery PLA324 hospital (Chongqing, China).Samples were snap frozen and stored in liquid nitrogen within 20 min after collection. All frozen tissues were confirmed as normal or cancerous by pathological examination. Specimens included 28 adenocarcinomas, 5 mucinous adenocarcinomas, and 3 mucinous adenocarcinomas complicated with adenocarcinoma. The number of high, moderate, and poor differentiated cases was 5, 18, and 13, respectively. All patients were diagnosed with stage II-III colorectal cancer by pathological examination. To avoid stromal contamination, samples were collected from the tumor center (greater than 1.5 cm), and adjacent tissues located 5 cm away from the colorectal cancer tissue were removed and considered as a normal control. The tumor absence was also confirmed by pathological examination(within 20 min). In this section, all 36 patients had tumors more than 12cm above the anal margin and none of them had neoadjuvant chemotherapy before operation.

The second section included 167 patients who were not the same as the 36 patients above. According to TNM system based on clinical and pathologic stage, all 167 patients were TNM stage III colorectal cancer(21 rectal cancer patients and 146 colon cancer patients), who did not receive neoadjuvant chemotherapy before operation, but received chemotherapy with oxaliplatin and 5-FU. Colorectal carcinoma tissues were collected from patients who underwent radical resection of colorectal cancer at the Department of General Surgery PLA324 hospital and Bethune International Peace Hospital. The tissue was preserved in 4% formaldehyde and was embedded in paraffin, and included 127 adenocarcinomas, 26 mucinous adenocarcinomas, and 14 mucinous adenocarcinomas complicated with adenocarcinoma. The number of high, moderate, and poor differentiated cases was 55, 53, and 58, respectively. Patients were followed for 60 months using hospital records and by telephone interviews with patients or families. Only colorectal cancer related deaths were considered to perform survival analysis. We obtained a complete follow-up on 126 men and 41 women. At the end of the study, 91 patients were cancer-free, 35 had tumor recurrence and 76 died because of cancer metastasis.

### Real-time PCR

Relative gene expression was measured in triplicate in the patients of the first section using TaqMan gene expression assay(Applied Biosystems) and 7500 real-time PCR system for the following gene transcripts: XPA (Invitrogen, Hs00166045_m1), XPC (Invitrogen, Hs01104206_m1), XPD (Applied Biosystems, Hs00361161), XPF (Applied Biosystems, Hs00193342), XPG (Applied Biosystems, Hs00164482), and ERCC1 (Applied Biosystems, Hs01012161). Gene expression was normalized to β-actin. To ensure that β-actin itself did not change between different samples, the ratio between β-actin and GAPDH, a second reference gene, was calculated. Relative gene expression was estimated using the 2^−ΔΔ^CT method as previously described (27).

### Western blot

Approximately 100 mg of colorectal tissue from the patients of the first section was homogenized in a glass homogenizer and lysed using 1 ml of precooled RIPA lysate for 15 s prior to incubation in an ice bath for 10 min. Cells were disrupted using ultrasounds (100 W for 5 s), centrifuged at 12,000 × g at 4°C for 10 min, and the supernatants were harvested. Total protein concentration was quantified using the Bradford method.

Fifty micrograms of protein were separated by sodium dodecyl sulfate polyacrylamide gel electrophoresis (SDS–PAGE) on a 12% gel, and proteins were transferred onto a polyvinylidene fluoride (PVDF) transfer membrane. The membrane was semidried at 20 V for 15 min, blocked with 5% skim milk for 4 h, washed three times of 5 min each with tris-buffered saline (TBS). Subsequently, goat anti-human XPC polyclonal antibody (Santa Cruz Biotechnology) (1:200) was added, and the membrane was incubated at 4°C overnight. After incubation with the primary antibody, a HRP-conjugated rabbit anti-goat IgG (1:3,000) (Zhongshan Golden Bridge Biotechnology Co., Beijing, China) was added, and the membrane was incubated at room temperature for 2 h. The membrane was stained using an enhanced chemiluminescence (ECL) reagent (Pierce, USA) and imaged onto an X-ray film (Fuji film, Japan) by autoradiography. Quantity One Imagine System and analysis software (Bio-Rad, USA) were used to quantitatively analyze the specific strips. β-actin was selected as the internal control. The relative protein level was expressed as the ratio between XPC and β-actin densities.

### Cell culture

SW1463 and HCT116 cell line were purchased from American Type Culture Collection (LGC-Promochem, Wiesbaden, Germany). And stored at −70°C. Cells were routinely cultured in Dulbecco's modified Eagle's medium (DMEM) (a high glucose medium) (Gibco, CA, USA) containing 10% fetal bovine serum (FBS) and incubated at 37°C in a 5% CO_2_ incubator. Cells were passaged every 2–3 days.

### Plasmid construction for SiRNA targeting XPC in colorectal cancer cells

An effective targeted versican sequence (5′-GGATGAAGCCCTCAGCGAT-3′) was screened from GenBank (No. giNM_004628, available at *www.pubmed.com*). The oligonucleotide chains were designed as a template based on the base pairing rule.

The following nucleotide sequences were used: forward (5′-GATCCGGATGAA-GCCCTCAGCGATTTCAAGAGAATCGCTGAGGGCTTCATCCTTTTTTGGAA-3′) and reverse (5′-AGCTTTTCCAAAAAAGGATGAAGCCCTCAGCGATTCTCTTGAA ATCGCTGA -GGGCTTCATCCG-3′). We also selected the following control sequences: forward (5′-GATCCGGATGAAGCCCTCAGCGATTTCAAGAGAGTGCACCGAGTCCTTCTGTATTTTTGGAAA-3′) and reverse (5′-AGCTTTTCCAAAAAATTACAGAAGGACTCGGTGCACTCTCTTGAAATCGCTGAGGGCTTCATCCG−3′). The oligonucleotides were synthesized by Invitrogen Co. (Shanghai, China).

The pSilencer™ 5.1-H1 Retro Vector (Ambion, No. AM5784) was digested using the restriction enzymes *Hind III* and *BamH I* followed by ligation with T4 DNA ligase. Then, the recombinant DNA was transformed into fresh competent *E. Coli* DH5α cells. The recombinant clones were picked from a solid Luria-Bertani (LB) broth plate containing 100 μg/ml ampicillin. The positive clones were confirmed by PCR and sent to the Shanghai GeneChem Company for sequencing. The confirmed efficient vector was called pSilencer™ 5.1-XPC siRNA, and the corresponding control vector was called pSilencer™ 5.1-XPC control. Lipofectamine™ 2000 was used to transfect SW1463 cells with the pSilencer™ 5.1-XPC siRNA and pSilencer™ 5.1-XPC control. Additional puromycin (1 μg/ml) was added to select the positive clones.

### Stable transfection of colorectal cancer cells with the pcDNA3-XPC plasmid

The pXPC-3 plasmid, carrying the XPC gene cDNA, was kindly donated by Junlei Zhang (Microbiology and Immunology Department of the third military medical university). A 3.4-kb DNA fragment containing the XPC gene cDNA was removed from the pXPC-3 plasmid DNA by Sfi I digestion and inserted into the Sfi I site of the pcDNA3.1(+) (Invitrogen) to obtain the pcDNA3-XPC plasmid. SW1463 cells and HCT116 cells were seeded in 100-mm cell culture dishes with 5 ml DMEM and cultured until a confluence of 70–80% was reached. Cells were transfected with pcDNA3-XPC plasmid DNA using the cationic lipid Lipofectamine® 2000 transfection reagent (10 μg plasmid DNA/50 μl Lipofectamine® 2000/100-mm dish) and incubated for 6 h. Cells were also transfected with pcDNA3 as a negative control using the same protocol.

### Immunohistochemistry

Immunohistochemistry was performed according to procedures previously described (28).Tissue sections from patients of the clinical trial second section, 5 μm thick, were deparaffinized, rehydrated in graded alcohols, and processed using the streptavidin immunoperoxidase method. In brief, sections were subjected to antigen retrieval by microwave oven treatment for 10 min in 0.01 mol/L citrate buffer (pH 6.0). Slides were subsequently incubated in 10% normal serum for 30 min, followed by an overnight incubation at 4°C with the appropriately diluted primary antibody. Mice anti-human monoclonal antibody was used at a 1:100 dilution. Subsequently, samples were incubated with biotinylated anti-mice or anti-rabbit immunoglobulins for 15 min at 37°C, followed by streptavidin peroxidase complexes for 15 min at 37°C. 3.3′-diaminobenzidine was used as the chromogen, and hematoxylin was used as a nuclear counterstain.

Immunohistochemical evaluation was conducted by at least two independent observers that scored the estimated percentage of tumor cells showing nuclear staining, independently of signal strength. An arbitrarily defined 15% cutoff was used to classify the colorectal carcinoma data into categorical groups (positive vs. negative).

### Cell susceptibility assay

SW1463 cells and HCT116 cells (1 × 10^6^/ml) were seeded in a 96-well plate (100 μl/well), and each treatment was performed in triplicate. Cells were either exposed toionizing radiation(IR) at different doses (0,1,2,4 Gy) or treated with cisplatin at different concentrations(0, 5, 20, 40 umol/L) (Sigma Company, Shanghai, China) for 4 h prior to evaluate their viability by 3-[4,5-dimethylthiazol-2-yl]-diphenyltetrazolium bromide (MTT). Cell viability was measured at 4 h after MTT addition (0.12 mg/ml) to assess the sensitivity to radiotherapy and chemotherapy. The absorbance was measured at 492 nm using a microplate reader (Bio-Rad, USA).

### Cell apoptosis assay by FACS

Annexin-V-FITC apoptosis assay kit was purchased from Baosai Biological Technology Co., Ltd. (Beijing, China). SW1463 cells and HCT116 cells were treated with IRat different doses (0, 1, 2, 4 Gy) or cisplatin at different concentrations (0, 5, 20, 40 umol/L) for 4 h prior to digestion with 0.1% trypsin. The cell suspension was centrifuged at 1,000 rpm for 5 min, the supernatant was removed and the cell precipitate was washed twice with PBS.

Next, 100 μlAnnexin-V-FITC was added to the cell precipitate and cells were incubated for 10–15 min at room temperature without light. Cells were centrifuged at 1,000 rpm for 5 min and washed once with PBS. Cell apoptosis was detected using a FACScan Flow Cytometer (Becton Dickinson, USA), and data were analyzed using CellQuest 3.0 software (Becton Dickinson, USA).

### Statistical analysis

SPSS 18.0 statistics software (SPSS Science, Chicago, USA) was used to perform the statistical analysis. All data are expressed as mean ± standard deviation. Each experiment was repeated at least three times, and all data represent the mean of at least three parallel samples. Analysis of variance (ANOVA) and Student's *t*-test were used to compare the differences among groups and between two groups, respectively. A contingency table was generated with the chi-square or Fisher exact probability test for immunohistochemistry data. The overall survival probability was calculated by the Kaplan-Meier method with statistical differences evaluated by the log rank test. The relative death risk of colorectal cancer was estimated by a multivariate Cox proportional hazard model. A *P* < 0.05 was considered statistically significant.

## Results

### Both XPC mRNA and protein expression were significantly upregulated in colorectal cancer tissues

**Figure 1:**
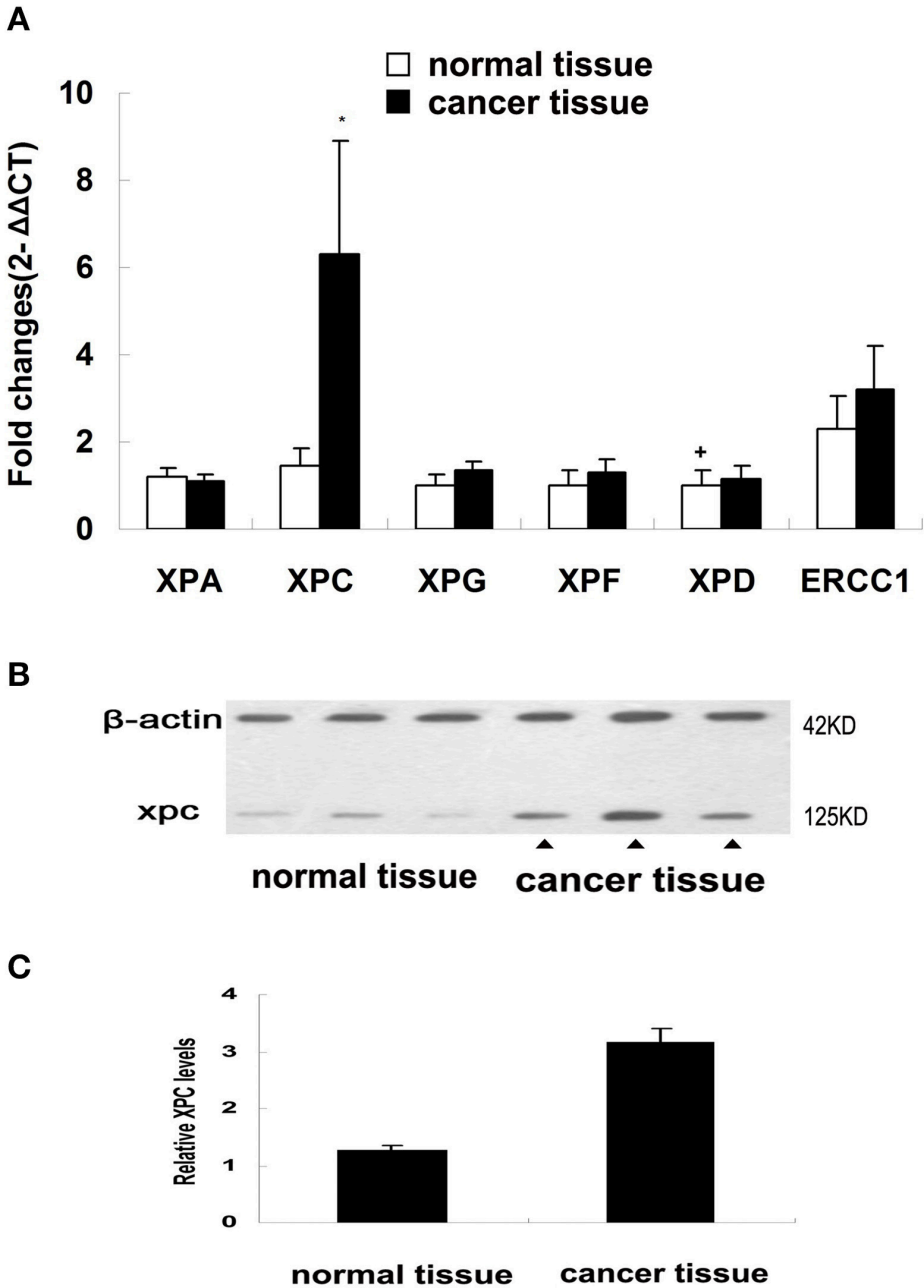
NER genes mRNA expression and XPC protein expression in normal colorectal tissue and colorectal tumor tissue. **(A)** NER gene transcripts in colorectal tumor tissues detected by real-time PCR. Changes were normalized against GAPDH, and all genes were compared with XPD (The mark of ‘+’ representing the expression of XPD in normal colorectal tissue as baseline). *P < 0.01 compared with the normal colorectal tissues. **(B)** XPC protein expression by western blot in colorectal tumor tissue and normal colorectal tissue of four patients. **(C)** XPC protein relative amounts in colorectal tumor tissue and normal colorectal tissue.

NER genes, such as XPA, XPC, XPG, XPF, XPD, and ERCC1, were measured in normal and cancerous human colorectal tissues belonging to the first section of the clinical trial and the results showed that only XPC mRNA level in the colorectal cancer tissues was significantly increased compared with the corresponding normal tissue (*P* < 0.01). No significant differences in XPA, XPD, XPG, XPF, ERCC1 mRNAs was found between colorectal cancer and normal tissues (Figure 1). XPC protein expression was also measured in normal andcolorectal cancer tissues. Western blot results revealed that XPC protein appeared at the predicted site of 120 kDa and its expression in the colorectal cancer tissue was significantly higher than its expression in the normal tissue (*P* < 0.05) (Figure 1).

### Different susceptibility of colorectal carcinoma cells to IR or cisplatin after XPC knockdown or overexpression

**Figure 2:**
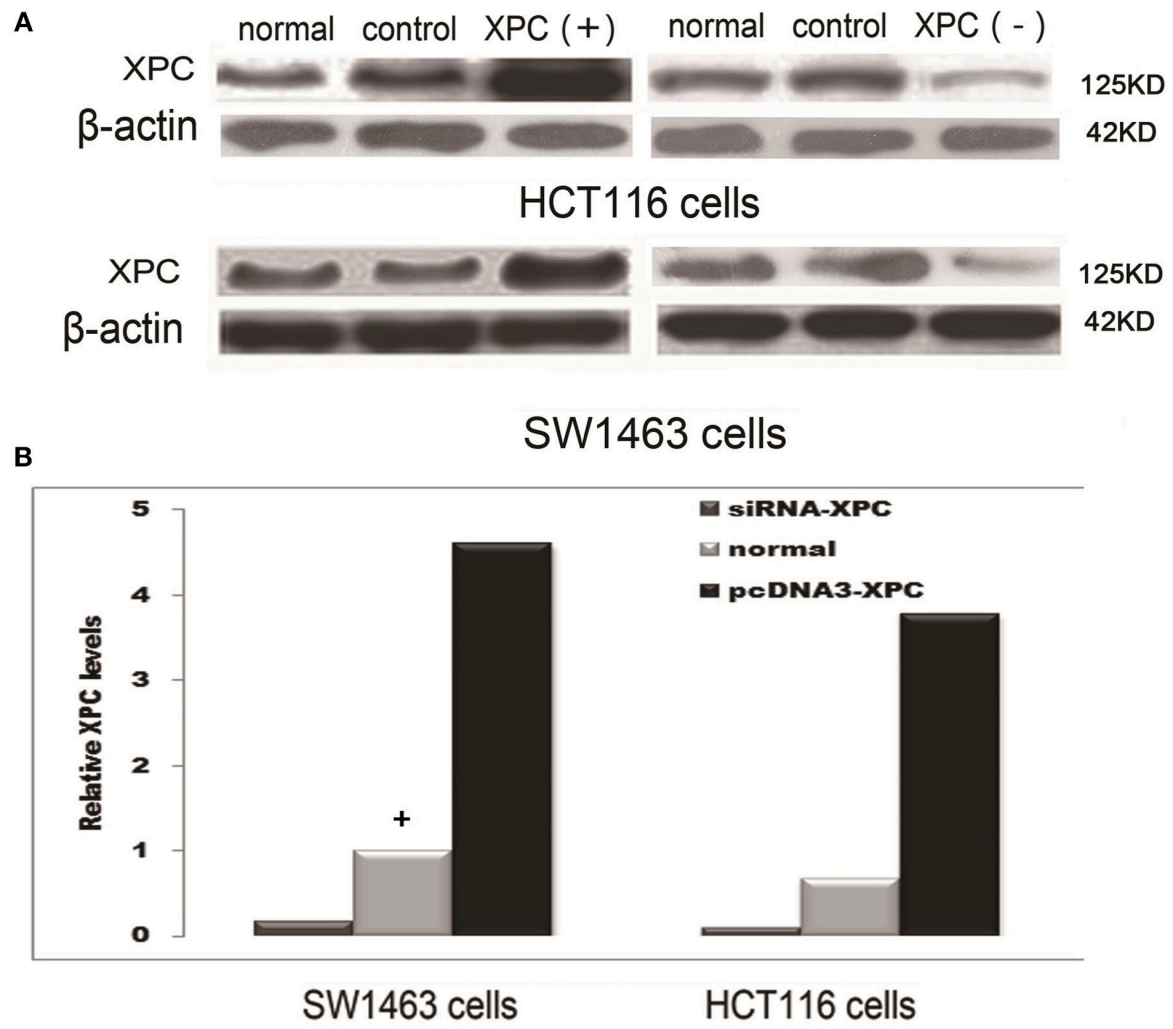
XPC protein expression in SW1463 and HCT116 cells prior to and after transfection with XPC siRNA or pcDNA3-XPC. **(A)** XPC protein expression by western blot in SW1463 and HCT116 cells. The top image shows a blot that was probed with XPC antibody, while the bottom image shows a blot that was probed with β-actin antibody. **(B)** XPC relative expression in SW1463 and HCT116 cells. XPC expression in the XPC silenced cells was significantly lower than that in the normal SW1463 and HCT116 cells (*P* < 0.01), whereas XPC expression in the pcDNA3-XPC-transfected cells was significantly higher than that in the normal SW1463 and HCT116 cells (Define the column with a “+” as baseline, the control group transferred empty plasmid into the cells) (*P* < 0.01).

**Figure 3:**
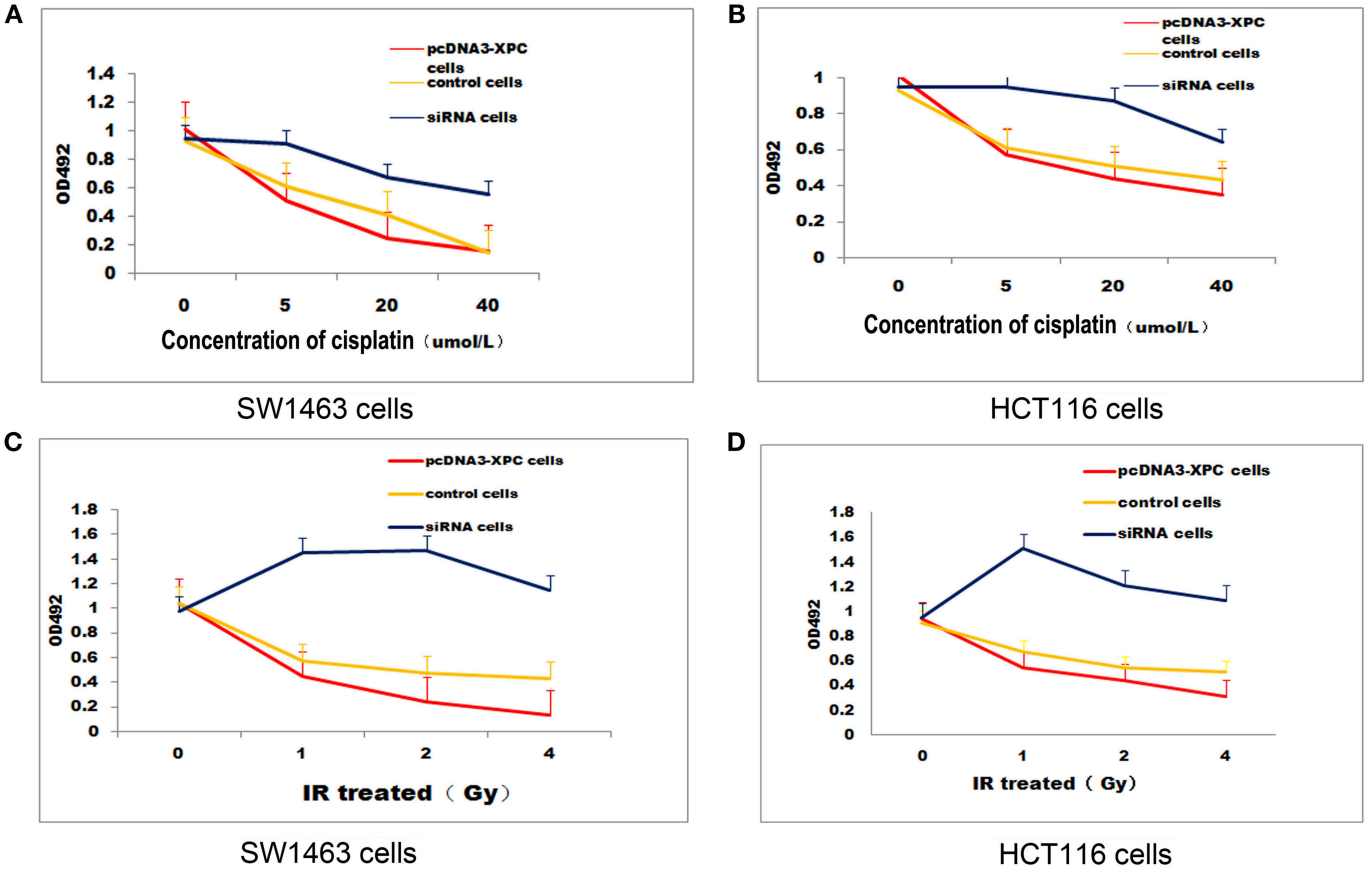
XPCoverexpression suppressed cell growth in the presence ofIR or cisplatin. IR and cisplatin growth inhibition was evaluated in pcDNA3-XPC transfected cells and XPC silenced cells. pcDNA3-XPC transfected cell were more susceptible to IR radiation or cisplatin compared to control cells and siRNA cells. **(A)** Control SW1463 cells, pcDNA3-XPC transfected SW1463 cells and XPC silenced SW1463 cells were exposed to cisplatin at different concentrations, followed by MTT analysis after 4 h. **(B)** Control HCT116 cells, pcDNA3-XPC transfected HCT116 cells and XPC silenced HCT116 cells were exposed to cisplatin at different concentrations, followed by MTT analysis after 4 h. **(C)** Control SW1463 cells, pcDNA3-XPC transfected SW1463 cells and XPC silenced SW1463 cells were exposed to IR at different intensities, followed by MTT analysis. **(D)** Control HCT116 cells, pcDNA3-XPC transfected HCT116 cells and XPC silenced HCT116 cells were exposed to IR at different intensities, followed by MTT analysis.

XPC siRNA significantly downregulated XPC protein expression in SW1463 and HCT116 colorectal cancer cell line, whereas XPC protein expression was significantly upregulated in the cells transfected with pcDNA3-XPC (Figure 2). IR or cisplatin significantly suppressed the growth of normal SW1463 and HCT116 cells (*P* < 0.05). Furthermore, XPC siRNA significantly reduced cell sensitivity to IR or cisplatin (*P* < 0.05) (Figure 3). However, pcDNA3-XPC-transfected cells became sensitive to cisplatin compared with siRNA cells under the same cisplatin treatment (*P* < 0.05). These results revealed an important role of XPC in the radiotherapeutic and chemotherapeutic susceptibility of colorectal cancer cells.

### Different apoptotic rate after IROR cisplatin treatment in XPC overexpressing or silenced SW1463 and HCT116 cells

**Figure 4:**
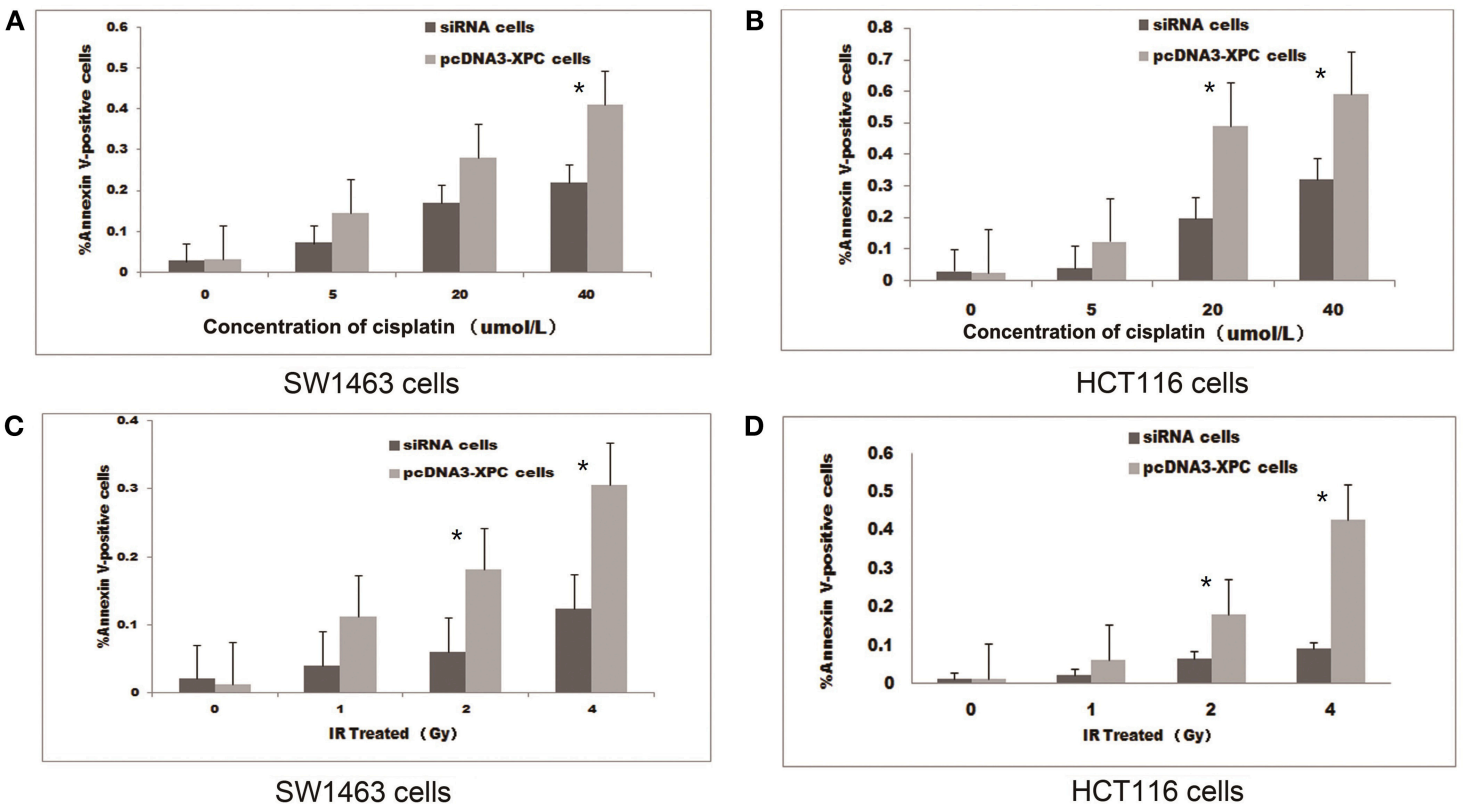
XPC overexpression increased cell apoptosis in the presence of IR or cisplatin. The apoptotic effect of IR and cisplatin on pcDNA3-XPC transfected cells and XPC silenced cells. Quantitative data are shown for annexin V-stained pcDNA3-XPC transfected cells and XPC silenced cells after treatment with cisplatin at different concentrations **(A,B)** and IR at different intensities for 4 h **(C,D)**. The percentage of apoptotic pcDNA3-XPC transfected cells was significantly higher than in normal cells (*P < 0.01) and XPC silenced cells (*P < 0.01).

The treatment with IR or cisplatin significantly increased the apoptosis of SW1463 and HCT116 cells (*P* < 0.01 and *P* < 0.05, respectively). Interestingly, the transfection with pcDNA3-XPC increased the percentage of apoptosis induced by cisplatin treatment or IR (*P* < 0.05).When SW1463 and HCT116 cells were transfected with XPC siRNA, the percentage of cell apoptosis was significantly decreased after cisplatin treatment (Figure 4). FACS results revealed that XPC overexpression promoted SW1463 and HCT116 apoptosis after cisplatin treatment or IR, whereas XPC silencing suppressed apoptosis, demonstrating a role of XPC in the radiotherapeutic and chemotherapeutic resistance of colorectal cancer.

### Correlation between XPC expression and colorectal cancer development

**Figure 5:**
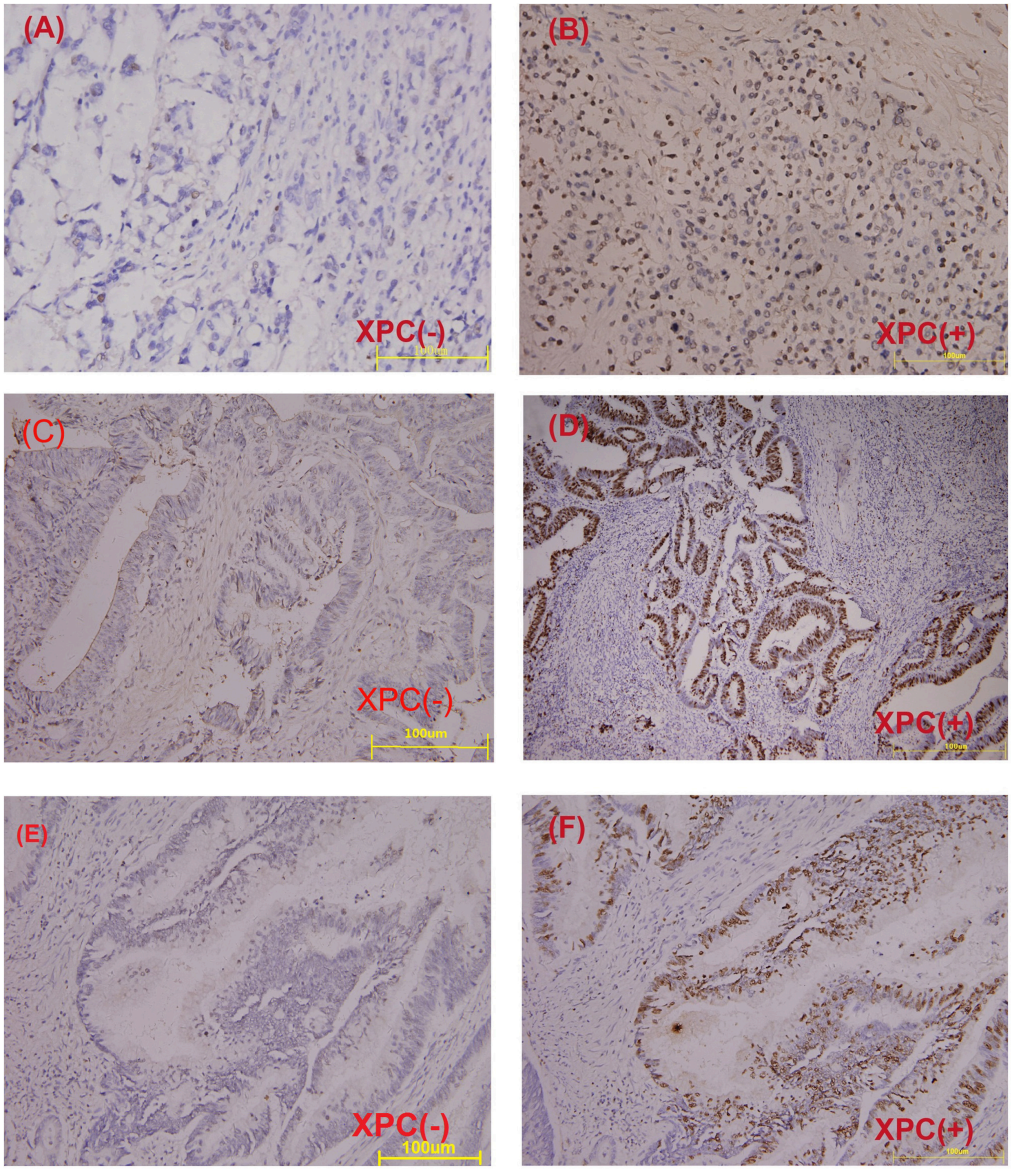
Immunohistochemical staining of paraffin embedded colorectal carcinoma**. (A)** Negative XPC expression in poor differentiated carcinoma. **(B)** Positive XPC expression in poor differentiated carcinoma. **(C)** Negative XPC expression in moderately differentiated carcinoma. **(D)** Positive XPC expression in moderately differentiated carcinoma. **(E)** Negative XPC expression in well differentiated carcinoma. **(F)** Positive XPC expression in well differentiated carcinoma.

The relationship between XPC expression and the development of colorectal cancer was analyzed in the second section of the clinical trial. XPC (+) expression was 73.2% in well differentiated cancer tissue, 62.3 and 46.6% in moderately differentiated and poor differentiated cancer tissue, respectively (Table 1 and Figure 5). Statistical analysis on immunohistochemistry data suggested that XPC (+) expression was significantly higher in well differentiated cancer tissue than poor differentiated cancer tissue (*P* < 0.05) (Table 1).

**Table 1:** Association of XPC expression with tumor differentiation.

	**XPC(+)**	**Total tumors**
**GRADE**		
Well differentiated carcinoma	41	56
Moderately differentiated carcinoma	33	53
Poor differentiated carcinoma	27	58
*P*-value	*P*^a^ = 0.151 *P*^b^ = 0.004 *P*^c^ = 0.071	

*P^a^-value is obtained by comparison between the group of well differentiated carcinoma and the group of moderately differentiated carcinoma; P^b^-value is obtained by the comparison between the group of well differentiated carcinoma and the group of poor differentiated carcinoma; P^c^-value is obtained by the comparison between the group of moderately differentiated carcinoma and the group of poor differentiated carcinoma*.

### Correlation between XPC expression and 5 year survival rate

**Figure 6:**
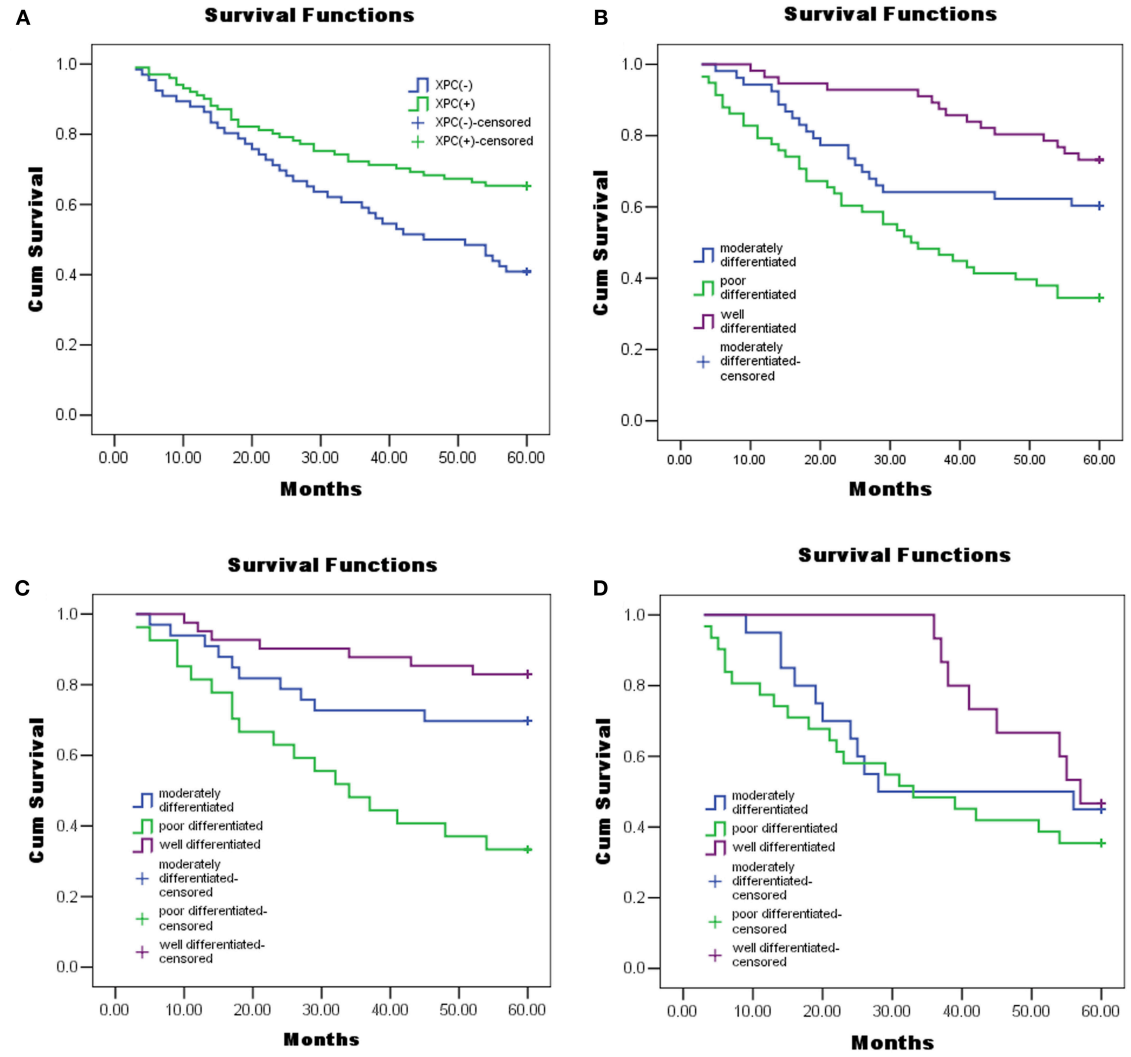
XPC expression and colorectal cancer patients' overall survival. Kaplan–Meier Curves using log rank tests for the overall survival of colorectal cancer patients was evaluated. **(A)**The overall survival of colorectal cancer patients with XPC(−) and XPC(+). **(B)** The overall survival of colorectal cancer patients with different degrees of differentiation. **(C)** The overall survival of colorectal cancer patients with high XPC expression in different degrees of tissue differentiation. **(D)** The overall survival of colorectal cancer patients with attenuated XPC expression in different degrees of tissue differentiation. Cum, cumulative.

Figure 6 shows the Kaplan-Meier curves of XPC with the adjusted *P*-values. The 5 year survival rate of patients with high XPC expression was significantly higher than that of patients with low XPC expression. However, the degree of tumor differentiation could significantly affect patient's survival time. Indeed, patients with high XPC expression had a significantly longer 5 year survival time than patients with low XPC expression in both well differentiated and moderately differentiated tumors (*P* < 0.01). However, this difference was not clear in the low differentiated group. Among the clinicopathological variables, tumor grade resulted as a significant prognostic factor after univariate analysis (Table 2). The multivariate analysis by Cox was performed, including all the variables significantly associated with survival at the univariate analysis and indicated that XPC expression was an independent factor predicting patients' outcome (Table 3).

**Table 2:** Univariate analysis of the influence of clinical characteristic on survival time in 167 patients with colorectal cancer.

**Parameter**	**No.patients**	**Median survive months**	***P*-value (log rank test)**
Gender:			0.878
Women	41	44.90	
Men	126	44.4	
Age:			0.495
60 or less	91	43.77	
More than 60	76	45.5	
Pathological grade:			0.000
Well differentiated	56	53.73	
Moderately differentiated	53	44.76	
Poor differentiated	58	35.52	
XPC:			0.003
Positive	101	47.23	
Negative	66	40.40	

**Table 3:** Cox multivariate regression analysis of potential survival time predictive factors in patients with colorectal cancer.

**Variable**	**Category**	**RR(95% CI)**	***P*-value**
Age	Less than 60/60 or more	1.143(0.709–1.842)	0.583
Gender	Men/Women	1.501(0.709–1.842)	0.145
Grade	Well/Moderately/Poor differentiated	0.213(0.113–0.403)	0.000
XPC	(−)/(+)	1.716(1.055–2.791)	0.030

## Discussion

Our study found that XPC expression in colorectal cancer tissue was significantly higher than that in the normal tissue and XPC overexpression increased the susceptibility of cells to cisplatin and IR in an *in vitro* experiment. Therefore, our study preliminarily proved that XPC might play an important predictiverole in the progression of colorectal cancer, although the mechanism is not yet clear.

Tumoroccurrence, development and meta stasisis a multi-step, multi-link, multi-faceted, multi-molecular process involving the activation of oncogenes and the inactivation of tumor suppressor genes (29), and the same molecule may play a different role at different stages of the tumorigenesis. Our study confirmed that the expression of XPC was closely related with the degree of differentiation of colorectal cancer. In addition, the expression of XPC in the highly differentiated colorectal cancer tissues was markedly higher than that in the poorly differentiated cancer tissues. The 5 year survival rate of patients with high XPC expression was significantly higher than that of patients with low XPC expression. Our results were similar to the results of previous studies that found that low XPC expression can promote tumor progression. Chen Z et al. (28) found a close relationship between low XPC expression and the occurrence of bladder cancer. Hosseini M et al. (30) suggested that low XPC expression facilitates the accumulation of oxidative damage, which in turn can cause tumorigenesis. All these evidences suggest that XPC, as a DNA damage recognizer, may play a protective role in the development of carcinogenesis.

However, once the tumor occurs, the DNA repair mechanism may lead to cancer drug resistance and poor prognosis by repairing the DNA damage induced by chemotherapeutic drugs. Fautrel et al. (31) found that the expression of XPC in hepatic carcinoma tissue was significantly higher than that in normal hepatic tissue, and they speculate that the high XPC expression may lead to drug resistance of the liver tumor by enhancing DNA repair.The role of XPC in different tumors may be related to the mutation of the p53 gene. Yang et al. (27) further demonstrated that low XPC expression contributes to p53 mutation in bladder cancer tissues, both promoting tumorigenesis. Some studies suggest that XPC can facilitate apoptosis in the presence of normal p53 and such a role was not observed in mutated p53 cells (32). Moreover, other studies revealed that NOX-1 overexpression enhances XPC ability to recognize damage in the absence of p53 (30).Furthermore, high XPC expression promotes apoptosis by inhibiting the expression of the anti-apoptotic protein Cas-2S,no matter whether p53 was mutated or not. Our study found that high XPC expression could improve the prognosis of patients with colorectal cancer by enhancing apoptosis induced by chemotherapeutic drugs. However, the study of Lin LC et al. (33) indicates that p53 mutation rate in colorectal cancer is ≥50% and p53 mutation results in poor prognosis of colorectal cancer patients. Thus, the link between XPC expression and p53 mutation needs further study.

Our study also found that high XPC expression enhanced cell sensitivity to IR. This effect might be due to the fact that XPC is not only a NER core protein but it is also involved in several other biological pathways, such as cell apoptosis, cell cycle regulation, oxidative damage recognition and base excision repair (30, 34, 35). The high XPC expression in colorectal cancer might be involved in these mechanisms and leading to a better prognosis. IR always induces apoptosis by damaging double strand DNA. Despras et al. (35) showed that low XPC expression weakened the double-stranded DNA injury repair capability of cells, while XPC overexpression enhanced such capability. Shell et al. (36) observed that high XPC expression occurs not only in DNA damages that can be repaired by NER but also in other types of DNA damages (e.g., DNA double-strand damage and oxidative damage).

Our results showed that even when transferring empty plasmid into the cells, the expression of XPC in SW1463 cells and HCT116 cells slightly increased. Our hypothesis is that this phenomenon might be related to the DNA recognition function of XPC, and unknown genes sequences of the plasmid transfected into the cell might induce the expression of XPC.

According to these studies, XPC participates in the recognition of DNA damages induced by chemotherapeutic drugs and initiates the subsequent apoptosis (severe DNA damages) or repair (mild DNA damages). Furthermore, the expression and function of XPC is regulated by many factors including p53 and NOX-1. Therefore, the pro-apoptotic role of XPC overexpression in colorectal cancer tissues might be affected by a variety of factors (e.g., the dose of chemotherapeutic drugs and p53 mutation) during chemotherapy or radiotherapy, although this aspect needs to be further explored. However, XPC is involved in several processes including cell cycle regulation, cell apoptosis and DNA damage repair, which undoubtedly provides new evidences in the occurrence, development and multi-drug resistance of colorectal cancer.

## Author contributions

JL and PY conceived and designed the experiments; L-BH, YC, and XH performed the experiments; X-DM contributed reagents, materials, and analysis tools.

### Conflict of interest statement

The authors declare that the research was conducted in the absence of any commercial or financial relationships that could be construed as a potential conflict of interest.
